# Changed categorical perception of consonant–vowel syllables induced by transcranial direct current stimulation (tDCS)

**DOI:** 10.1186/s12868-016-0241-3

**Published:** 2016-02-01

**Authors:** Kai Heimrath, Anna Fischer, Hans-Jochen Heinze, Tino Zaehle

**Affiliations:** Department of Neurology, Otto-von-Guericke University Magdeburg, Leipziger Street 44, 39120 Magdeburg, Germany

**Keywords:** tDCS, Auditory cortex, Auditory temporal perception, Voice onset time, Categorical perception

## Abstract

**Background:**

Speech-related disorders may refer to impairment of temporal analysis in the human auditory system. By the advance of non-invasive brain stimulation new forms of therapy arise. In the present study, we examined the neuromodulatory effect of auditory tDCS on the perception of temporal modulated speech syllables. In three experimental sessions we assessed phonetic categorization of consonant–vowels (CV)-syllables (/da/,/ta/) with varying voice onset times (VOT) during sham, anodal, and cathodal tDCS delivered bilateral to the auditory cortex (AC). Subsequently, we recorded auditory evoked potentials (AEP) in response to voiced (/ba/,/da/,/ga/) and voiceless (/pa/,/ta/,/ka/) CV-syllables.

**Results:**

In result, we demonstrate that bilateral tDCS of the AC can modulate CV-syllable perception. Behaviorally, cathodal tDCS improved phonetic categorization abilities in a VOT continuum accompanied by an elevation of the P50 amplitude of the AEP to CV-syllables during the anodal tDCS after effect.

**Conclusions:**

The present study demonstrates the ability of bilateral tDCS over the AC to ameliorate speech perception. The results may have clinical implications by fostering potential approaches for a treatment of speech-related pathologies with a deficit of temporal processing.

## Background

Speech perception requires the recognition and discrimination of phonemes, in particular the encoding of temporal information in short linguistic elements such as consonants and vowels. A main feature to categorize stop-consonants is the voice onset time (VOT), which is defined as the duration of the delay between release of closure and start of voicing. It characterizes voicing differences in a variety of languages and distinguishes voiced stop consonants (/b/,/d/,/g/) from their voiceless counterparts (/p/,/t/,/k/) [1]. Discriminating voiced and unvoiced syllables in a consonant–vowel (CV)-VOT continuum is categorical by exhibiting two qualitatively discrete percepts. The neuronal activity of the auditory cortices during the processing of different VOT’s in speech stimuli is reflected by the P50-N1 complex of the auditory evoked potential (AEP) [2–6]. Accordingly, the P50-N1 complex has been successfully shown to reflect neural representation of feature processing of the acoustic stimulus [7, 8].

Speech related disorders have been associated with altered acoustic processing abilities. Children with general language-learning disabilities [9, 10] and children and adults with dyslexia [11, 12] show an impaired auditory processing of temporal information during speech perception. Specifically, these patients demonstrated deficient phoneme perception abilities, reflected by inconsistent labeling of CV-syllables in a VOT continuum [13–16].

As a completion to conventional approaches that treat temporal processing deficits in dyslexics by perceptual training [17–20], transcranial direct current stimulation (tDCS) might be a promising therapeutic tool. During tDCS low currents are delivered to the cerebral cortex resulting in a modulation of cortical excitability [21]. The current flows between an active and a reference electrode through the skull to the brain tissue, thereby inducing diminutions or enhancements of cortical excitability [22]. The direction of the tDCS-induced effect depends on the current polarity. Anodal tDCS typically increases and cathodal tDCS decreases the cortical excitability in the region under the electrode.

Given the neuromodulatory potential of tDCS to alter auditory cortex (AC) reactivity [23, 24] as well as spectro-temporal perception [25–27], in the present study, we investigated the effects of tDCS over the bilateral temporal cortex on phonetic categorization of CV-syllables in a VOT continuum. We hypothesized tDCS-dependent alterations in the performance of a phonetic categorization task. Furthermore, we recorded and compared AEPs in response to voiced and voiceless CV-syllables after tDCS application and expect tDCS induced changes in the neuronal reactivity of the AC reflected by modulations of the P50-N1 complex.

## Results

### Behavioral data

**Fig. 1:**
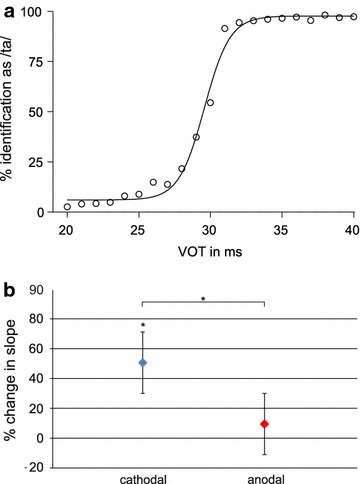
**a** Subjects performance on phonetic categorization averaged across tDCS conditions (sham, anodal, and cathodal). The *graph* indicates the percentage of CV-syllables that were identified as/ta/in relation to their VOT (*circles*) and the logistic curve fit. **b** Effect of active tDCS on phonetic categorization. Individual changes in slope are plotted relative to normalized sham condition (Mean ± SEM)

Figure 1a shows the averaged CV-syllable identification curve for the percental /ta/ identification illustrating that, overall, participants successfully categorized phonetic stimuli. The analysis revealed a mean slope parameter (β1) for tDCS conditions (sham = 1.23, SE ± 0.14; anodal = 1.31, SE ± 0.27; cathodal = 1.74, SE ± 0.27). As shown in Fig. 1b, cathodal tDCS steepened the slope parameter (β1) of the identification curves by 50 % compared to sham baseline performance (t(1,12) = 2.387, p = 0.03). Furthermore, simultaneous cathodal tDCS had a significant stronger effect on β1 than anodal tDCS (t(1,12) = 2.53, p = 0.03). Simultaneous anodal tDCS caused no considerable changes from sham baseline performance (t(1,12) = 0.464, p = 0.65). Thus, concurrent cathodal tDCS improved the categorical perception of a CV-VOT continuum demonstrating the ability to sharpen phonetic perception by means of bilateral auditory cathodal tDCS.

### Auditory evoked potentials

**Fig. 2:**
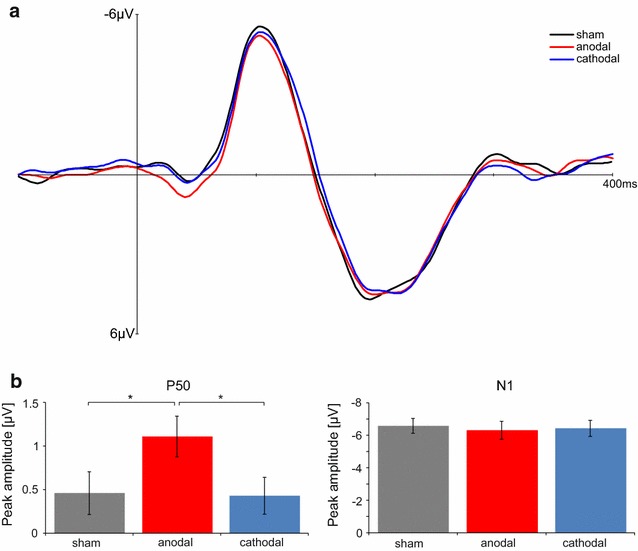
**a** Grand average AEPs recorded at channel Cz are shown for different conditions (sham, anodal, and cathodal). **b** P50 and N1 amplitudes recorded at channel Cz for different tDCS conditions (sham, anodal, and cathodal) (Mean ± SEM)

AEPs in response to CV-syllables are illustrated in Fig. 2. All stimuli evoked measurable P50 and N1 components. Repeated measures ANOVA with the factor *tDCS* (sham, anodal, cathodal) for P50 amplitude revealed a significant main effect [F(2,24) = 5.985, p = 0.01] due to significant larger P50 amplitude after anodal in contrast to sham tDCS (t(12) = 2.441, p = 0.03) and cathodal tDCS (t(12) = 3.676; p = 0.01). P50 amplitude after cathodal tDCS did not differ compared to sham tDCS (t(12) = 0.114; p = 0.89). For the N1 amplitude repeated measures ANOVA showed no tDCS effect [F(1,12) = 0.488, p = 0.62] (cf. Figure 2b). No differences in the P50(F(2,24) = 0.053, p = 0.95) and N1 latencies (F(2,24) = 2.037, p = 0.15) could be observed.

## Discussion

In the present study we demonstrate that phonetic perception can be modulated by bilateral tDCS of the AC. Categorization of CV-syllables in a VOT continuum was enhanced by cathodal tDC-stimulation. In particular, concurrent cathodal tDCS steepened the slope of the identification curve indicating more consistent categorization of the syllables/ta/and/da/. This sharpening of the phonetic perception was accompanied by increased P50 amplitude in response to natural CV syllables after anodal stimulation.

In our study, cathodal tDCS improved preciseness of phonetic categorization, with no influences of anodal tDCS. In a first attempt this result might be contradictive in the light of the polarity-specific dichotomy assuming that anodal tDCS typically improves while cathodal tDCS worsens the behavioral outcome in a specific task. Notably, these dual-polarity effects have mainly been demonstrated in the motor domain but less on cognitive functions [28]. Particularly in the auditory domain, several studies demonstrated a decrement of performance induced by cathodal tDCS on auditory function [25, 29], but there is also evidence for an opposite effect showing improved performance after cathodal stimulation [30]. It can be assumed that different stimulation parameter such as stimulation power, electrode size, and electrode placement especially of the reference electrode, as well as the individual auditory stimuli contribute to the varying tDCS-effects. Furthermore, the tDCS-related alterations of the neurotransmitter level may impact homeostatic plasticity in the auditory domain. Whereas anodal tDCS reduces local concentrations of the inhibitory neurotransmitter gamma-amino butyric acid (GABA), thus, inducing improvement, cathodal tDCS reduces excitatory glutamate levels followed by impoverishment of the behavioral outcome. However, there is also evidence that cathodal tDCS can decrease GABA concentration and thus may induce improved performance as well [31, 32]. Thus, given that regional cortical excitation/inhibition balance, measured by ratios of glutamate/GABA, provide meaningful interpretations of individual cognitive as well as perceptual performance [33], cathodal tDCS may artificially change the excitation/inhibition balance towards a more optimal level in the auditory cortex.

The present results extend the view of tDCS induced modulations on temporal processing by showing improved phonetic categorization of CV-syllables with varying VOTs. This might reflect a facilitation of low-level acoustic processing of temporal features in the AC. Moreover, we assessed the electrophysiological brain activity in order to investigate tDCS induced after effects on CV-syllable perception. It has been proposed that anodal tDCS over the temporal cortex can alter AC reactivity resulting in modulation of the AEPs. As has been demonstrated previously using sinus tones [23], we found enhanced P50 amplitudes after unilateral anodal tDCS indicating that alterations in the AC reactivity impact early stages of perceptual processing. Basically, the P50 component of the AEP presumably reflects sensory representation of an acoustic stimulus in the AC [34, 35]. Accordingly, uni- as well as bilateral anodal tDCS over the AC increases P50 amplitudes to acoustic stimuli indicating a general neuromodulatory effect on early sensory acoustic processing. Remarkably, the present study shows enhancement of the AC reactivity after anodal- but not cathodal tDCS. Such anodal tDCS-related increase in cortical excitation could be assumed to be the cause of an improved auditory performance. However, our behavioral data during tDCS showed no change of performance during anodal condition. Accordingly, we cannot directly relate the observed electrophysiological modulations after tDCS to the improved auditory phonetic categorization abilities described with concurrent stimulation. However, tDCS efficiency on cortical excitability critically depends on the timing of the stimulation. Several studies showed that tDCS can result in contradictive effects during (online) and after the application of tDCS (offline). For instance, simultaneous anodal tDCS leads to an improvement in motor learning and working memory performance, whereas during the after-effect anodal tDCS results in no or opposite effects [36–38]. Such opposite online vs. offline effects have been found for the visual domain showing improved perceptual learning after but not during cathodal tDCS [39]. Analogously, online tDCS decreased motor learning, whereas motor performance was worsened during the after-effect [36, 40]. These opposing effects might be related to the underlying physiological actions of online vs. offline tDCS. While acute-effects during stimulation (online) are primary based on changed membrane potentials, post-stimulation after-effects are related to NMDA-receptor activation indicating a LTP-like mechanism for learning [41–43]. Consequently, these differential underlying physiological actions during and after stimulation may lead to opposite effects of tDCS. One might further speculate that bilateral tDCS application over the AC might influence the mutual inhibition between the two hemispheres and thereby inducing opposite effects. Moreover, contradictive results might be also related to the different auditory stimuli used during and after tDCS application. While subjects heard ambiguous syllables from the VOT continuum, during EEG-recording non-ambiguous syllables were presented.

Our results demonstrate that simultaneous cathodal tDCS can induce an enhancement of auditory performance, whereas anodal tDCS induces after-effects that enhance AC reactivity.

Nevertheless, the present findings may have clinical implications for the treatment of speech-related pathologies such as dyslexia. Dyslectic children as well as adults exhibit deficits in the processing of rapid auditory information accompanied with deficient phonological processing [13, 14, 44]. Those patients may benefit from tDCS administration as add-on to conventional therapy. Notwithstanding the fact that the neurophysiological mechanisms are still not fully understood the current results show that tDCS can be successfully used to modulate rapid temporal processing of speech sounds. Consequently, by modulating the excitability of the temporal cortex via non-invasive brain stimulation, the present study provides a novel approach that can be simply administered to address stunted temporal processing abilities in auditory disorders in the human brain.

## Conclusion

To our knowledge this is the first study investigating tDCS effects on phonetic perception by behavioral and electrophysiological parameter. Our results show that bilateral tDCS of the temporal lobe can change the cortical reactivity and the performance associated with phonetic categorization. Additional studies are needed to provide a better understanding of the behavioral and neurophysiological basis of tDCS efficiency in the human AC.

## Methods

### Participants

13 human subjects (mean age 25.92 ± 3.15; 7 male) participated in this study. Participants gave written informed consent in accordance with the 2013 World Medical Association Declaration of Helsinki. All subjects were native German speakers and had no history of neurological, psychological or hearing impairment. All procedures were approved by the ethics committee of the University of Magdeburg.

### Transcranial direct current stimulation

All participants received on three different days one session of either bilateral sham, anodal or cathodal stimulation over the AC in a randomized order. The sessions were separated by at least 48 h to avoid carry over effects. TDCS was applied by a battery driven constant current stimulator (ELDITH, NeuroConn GmbH, Germany) using three rubber electrodes placed in 0.9 % saline-soaked synthetic sponges. Two 5 × 5 cm stimulation electrodes were placed over T7 and T8 according to the 10–20 system for EEG electrode placement. A 5 × 10 cm reference electrode was placed longitudinally over electrode site Cz. The stimulation electrode placement has been shown to modulate low-level processing and cortical reactivity in the AC [23, 25]. The direct current was applied with a strength of 1.5 mA and 10 s fade in/out. For sham condition, the stimulation was turned off after 30 s without the awareness of the participants with linear fade out time of 10 s. This procedure ensured that in the sham and stimulation conditions, participants experienced the initial itching that recedes over the first seconds of tDCS. Accordingly, none of the participants were able to reliably determine whether or not they received active or sham stimulation.

### Stimuli

The auditory stimuli were generated (sampling depth of 32 bits and a sampling rate of 44.1 kHz) using Software SoundForge 4.5 (Sonic Foundry Inc., 1999) and Praat (Version 5.3.63). The duration of each single stimulus was 330 ms. Stimulus presentation was controlled by the Presentation software (Neurobehavioral Systems, USA). The stimuli were presented binaurally via headphones (Sennheiser, HD 65TV) with a sound pressure level of 75 dB.

### Procedure

To familiarize the participants with the task, prior to every session participants practiced the phonetic categorization. Then tDCS application was started. After 10 min of consecutive tDCS, a phonetic categorization task (CV-task I) started, while tDC-stimulation continued. For the CV-task, a synthetic VOT continuum was used ranging from 20 to 40 ms VOT in 1 ms steps [2]. Participants were instructed to listen to each syllable and to decide whether the syllable was the voiced syllable/da/or the voiceless syllable /ta/ by pressing a corresponding button. Each of the 21 CV-syllable was presented 18 times in a randomized order. The task duration was 12 min. Subsequently, tDCS-electrodes were removed and EEG-electrodes were mounted. The time interval between the end of the tDCS and the start of the EEG session was 11.7 min ± 3.6 min. During the second task (CV-task II) AEPs were recorded in response to voiced (/da/,/ba/,/ga/) and voiceless (/ta/,/pa/,/ka/) natural CV-syllables. Participants had to decide whether the CV-syllable was voiced or voiceless by pressing the corresponding button. Each CV-syllable was presented 50 times in a randomized order with a delay time of 1000 ms after subjects’ response. Performance rate was equal above 97 % in all three stimulation conditions (sham 97.75 %, anodal 97.31 %, cathodal 98.1 %; F(2, 24) = 1.974, p = 0.161).

### EEG recording

EEG was recorded during CV-task II after tDC-stimulation from standard scalp locations Fz, Cz and Pz according to the international 10–20 system using Ag/AgCl electrodes mounted in an elastic cap. The electrooculogram was recorded with one electrode placed below and approximately 1 cm to the external canthus of the left eye. EEG data were recorded by a Brainamp DC amplifier (Brain Products) and the corresponding software (Brainproducts, Brain Vision Recorder 1.20) referenced to the right mastoid and sampled at 500 Hz. Impedances were kept below 5 kΩ.

### Data analysis

#### Behavioral data

To examine performance in the CV-task-I we analyzed the slope parameter (β1) of the individual identification curves. This parameter provides a reliable measure for the preciseness of categorical perception in a VOT continuum [13, 14, 16, 45], with high values of β1 indicating a steep increase of the identification curve and reflecting high preciseness in categorical perception, and low values of β1 denoting a shallow, more fuzzy categorical perception. For this, we fitted each individual identification curve with the following formula:

$$x(y) = \frac{1}{{(1 + e^{{( - \left( {\upbeta 1*x \,+\, \upbeta 0} \right)}} )}}$$

and calculated the individual category boundary x(y = 0.5), which is the point of 50 % correct responses or the point of maximal confusion. On average across the three tDCS conditions (sham, anodal, and cathodal) this point was found on a VOT of 29.2 ms (cf. Figure 1a). Subsequently, we extracted the individual slope parameter (β1) at this category boundary (VOT 29 ms ± 2 ms). For analysis, the categorization parameter was normalized to the individual data during sham condition (baseline) to control for inter-individual variance, and compared between stimulation conditions by means of paired-sample t-tests.

#### Electrophysiological data

EEG preprocessing and data analysis were carried out using EEGlab V.12 (http://www.sccn.ucsd.edu/eeglab/). EEG data were off-line filtered from 0.01 Hz to 40 Hz. Segments containing ocular artifacts, movement artifacts, or amplifier saturation were excluded from the averaged ERP waveforms. The EEG recordings were sectioned into 600 ms epochs (200 ms pre-stimulus and 400 ms post-stimulus) and a baseline correction using the pre-stimulus portion of the signal was carried out. ERPs in response to all CV-syllables were averaged for each subject and grand-averaged across subjects. A peak analysis was performed on single-subject averages measured at channel Cz, which showed the largest deflections in the grand average. AEPs were quantified by measuring the baseline-to-peak amplitudes for the most positive (P50) and negative peak (N1) occurring at specific latency ranges (P50 20–70 ms; N1 80–140 ms). In the end, amplitude of the P50 and N1 components were analyzed using separate repeated-measures ANOVAs with a within-subject factor *tDCS* (sham, anodal, and cathodal). For post hoc analysis paired samples t-tests were performed.

## Abbreviations

AC: auditory cortex
AEP: auditory evoked potential
ANOVA: analysis of variance
CV: consonant–vowel
LTP: long-term potentiation
tDCS: transcranial direct current stimulation
VOT: voice onset time
